# Safety and efficacy of biocompatible perfusion strategy in a contemporary series of patients undergoing coronary artery bypass grafting – a two-center study

**DOI:** 10.1186/s13019-014-0196-3

**Published:** 2014-12-18

**Authors:** Oz M Shapira, Amit Korach, Frederic Pinaud, Abeer Dabah, Yusheng Bao, Jean Jacques Corbeau, Jean-Louis de Brux, Christophe Baufreton

**Affiliations:** Department of Cardiothoracic Surgery, Hebrew University, Hadassah Medical Center, POB 12000, Ein-Kerem, 91120 Jerusalem Israel; Department of Cardiac Surgery, University Hospital of Angers, Angers, France

**Keywords:** Cardiopulmonary Bypass, Coronary Artery Bypass Grafting, Biocompatible, Clinical outcomes, Blood Transfusions

## Abstract

**Objective:**

The profile of patients referred for coronary artery bypass grafting (CABG) is continuously changing to include older patients with multiple comorbidities. We assessed the safety and efficacy of a biocompatible perfusion strategy (BPS) in a contemporary series of patients undergoing isolated CABG.

**Methods:**

BPS consisted of a membrane oxygenator, tip-to-tip closed-system heparin-bonded cardiopulmonary bypass circuits without a cardiotomy reservoir, low systemic anticoagulation (target ACT – 250-300 sec) using heparin titration curves, low prime volume, avoidance of systemic cooling, and routine use of cell saver and anti-fibrinolytics. Data were prospectively collected using the American Society of Thoracic Surgeons National Adult Cardiac Surgery Database definitions.

**Results:**

964 consecutive patients (mean age 66 ± 11 years, 83% male) undergoing CABG between 2008 and 2012 were enrolled. 30-day mortality was 1.4%. Rates of postoperative stroke, myocardial infarction, sternal infection and reoperation for bleeding were 0.9%, 1.3%, 1.9% and 4.2%, respectively. Average 24-hour chest tube drainage was 440 ± 280 ml. Blood products were used in 34% of patients (total donor exposure of 1.7 ± 4.7 units/patient). Predictors of hospital mortality in multivariable analysis were left main disease and preoperative treatment with anti-arrhythmic or immunosuppressive medications. Predictors of allogeneic blood transfusions included older age, small body surface area, female gender, increased serum creatinine, lower preoperative LVEF and hematocrit. Priority of surgery, dual antiplatelet therapy and cardiopulmonary bypass time were not predictors of adverse outcomes or blood transfusions.

**Conclusions:**

In a contemporary cohort of patients undergoing CABG, the use of BPS is safe and effective. It is associated with excellent clinical outcomes and reduced allogeneic blood transfusions.

**Electronic supplementary material:**

The online version of this article (doi:10.1186/s13019-014-0196-3) contains supplementary material, which is available to authorized users.

## Background

The use of cardiopulmonary bypass (CPB) in coronary artery bypass grafting (CABG) surgery affords the opportunity to achieve the most important goals of this operation – complete revascularization, and performing the anastomoses in an ideal setting of bloodless and motionless field – hence very precisely. These factors translate into clinical benefits of improved patient survival and reduced rates of cardiac events and coronary re-interventions compared to either percutaneous coronary intervention (PCI) or off-pump CABG [1],[2].

However, the use of CPB is associated with side-effects and complications inherent to the pathophysiology of this technology which involve a mechanical pump, blood-foreign surface interaction, blood-air interface and micro-embolization [3]. The end results include systemic inflammatory response, coagulopathy and organ dysfunction [3].

To attenuate these pathophysiological phenomena we and others developed a unique and comprehensive biocompatible perfusion strategy (BPS) in the mid 1990’s and have shown that indeed this strategy is safe and associated with improved clinical outcomes in patients undergoing CABG, valve and aortic operations [4]-[8].

Since these reports, however, the profile of patients referred for CABG has fundamentally changed to include older patients with multiple co-morbidities [9]. Patients often are referred in the setting of acute coronary syndrome on dual anti-platelet therapy and after one or more PCIs [10].

Given this substantially higher patient risk profile we aimed to reassess the safety and efficacy of our BPS in a contemporary cohort of patients undergoing CABG. The BPS was independently adopted by the University of Angers in the late 1990’s [11] and was introduced to Hadassah in the beginning of 2008. In this study we evaluate the experience of both institutions over the past five years.

## Methods

### Patients

964 consecutive patients undergoing isolated CABG using CPB between January, 2008 and June, 2012 were enrolled. Patients undergoing off-pump CABG or CABG with concomitant procedures were excluded. The study was approved by the Hadassah Hebrew University Medical Center and the University of Angers Helsinki Committees. The need for informed consent was waived by both committees since this was a retrospective study using unidentified data obtained from Departmental databases.

### Biocompatible perfusion strategy

The components of our biocompatible perfusion strategy have been previously described [4],[5]. Both institutions used an identical uniform protocol. The BPS was adopted by the entire faculty in Angers and two surgeons (out of four) at Hadassah. Briefly, it consisted of tip-to-tip, closed-system (collapsible closed venous reservoir) heparin-coated CPB circuits (Carmeda®, Medtronic, Minneapolis, MN, USA, or Bioline®, Maquet, Wayne, NJ, USA) with a membrane oxygenator and lack of a cardiotomy reservoir. This circuit configuration nearly eliminates the blood-air interface. Anticoagulation was carefully monitored using heparin titration curves (Hepcon®, HMS Plus, Medtronic, Minneapolis, MN, USA) with a target activated clotting time (ACT) of 250–300 sec. CPB prime volume was reduced to a minimum using the retrograde autologous priming technique [12]. An important safety practice is strict avoidance of blood stasis within the CPB circuits, i.e. – the volume of blood within a segment of circuit that has no flow must be replaced by a crystalloid solution. Systemic body temperature was kept at near-normothermia (34-36°C) avoiding active cooling. We routinely used a cell saver and anti-fibrinolytics (Tranexamic acid). The threshold for red blood cell transfusion was set at hematocrit of less than 20% during CPB and less than 25% after CPB. Persistent postoperative bleeding in excess of 300 mL in the first hour or 500 mL in the first two hours was considered to be an indication for platelet (5–10 units) and fresh frozen plasma (2 units), in the presence of supporting laboratory test results. We did not use thromboelastography. Routine postoperative pharmacological management included oral administration of 100 mg acetylsalicylic acid once daily, started on the first postoperative day, and subcutaneous injection of 1 mg/Kg of enoxaparin started on the first postoperative day and terminated at discharge. Patients with indwelling drug-eluting coronary stents implanted within one year before surgery were treated with oral 75 mg clopidogrel daily in addition to acetylsalicylic acid.

### Data collection and study endpoints

Data in both institutions were prospectively collected and entered into a Departmental database. Data collection was performed using the American Society of Thoracic Surgeons (STS) Adult Cardiac Surgery Database definitions using the STS collection tool version 2.73 [13]. Routine prospective data collection optimized two fundamental requirements – data completeness and accuracy. Data collected included baseline patient demographic and risk profile, operative data and 30-day clinical outcomes. The primary study endpoints included 30-day mortality and the incidence and magnitude of allogeneic blood transfusions. Secondary endpoints included rates of major complications including perioperative myocardial infarction, stroke, re-exploration for bleeding and sternal wound infection, time on the respirator and length of hospital and intensive-care unit stay. We focused on these endpoints to assess the safety of the BPS, particularly with regards to the risk of thromboembolic events secondary to low systemic anticoagulation. Bleeding and transfusion requirements were used as endpoints to assess the impact of the BPS on cardiopulmonary-bypass-associated coagulopathy. The STS Database criteria were used to define the primary and secondary endpoints [13].

### Statistical analysis

Continuous variables were expressed as mean ± standard deviation, median and range. Categorical data were expressed as absolute values with percentages. To examine the primary study- endpoints we performed univariable and multivariable analyses. Continuous variables were analyzed using the Student t test. Categorical data were analyzed using the X^2^ with Yate’s correction. Variables with a P value of ≤0.02 were entered into stepwise multiple logistic regression models to determine independent predictors of primary study endpoints. Results were expressed as odds ratios (OR) with 95% confidence intervals (CI). A P value of 0.05 was considered significant. Statistical analyses were performed using the SPSS for Windows, Version 18.0 software.

## Results

### Patients

There were 964 patients (males – 804 {83%}, females – 160 {17%}) with a men age of 66 ± 11 years (range 32 to 89 years). The patients’ baseline profile is depicted in Table 1. It reflects the typical profile of patients referred for CABG nowadays. Notably, 36% of patients were diabetics, 40% had left main disease, 57% had prior MI, 29% had prior PCI and many had been on aspirin, a second antiplatelet agent or heparin.

**Table 1:** **Baseline patient profile**

Variable	Percent or mean, SD (Median, Range), (n = 964)
Age (Years)	66 ± 11 (66, 32–89)
Gender (M/F)	804 (83%) / 160 (17%)
Hypertension	694 (72%)
Diabetes mellitus	345 (36%)
Peripheral vascular disease	184 (19%)
Prior cerebrovascular accident	62 (6%)
Renal failure on dialysis	13 (1.3%)
Chronic obstructive pulmonary disease	90 (9%)
Prior myocardial infarction	556 (58%)
Prior Percutaneous Coronary Intervention	282 (29%)
Preoperative intra-aortic balloon pump	42 (4.3%)
Preoperative medications	
Aspirin	878 (91%)
Second antiplatelet agent within 5 days	36 (4%)
Heparin	342 (35%)
Anti-arrhythmic medication	32 (3%)
Immunosuppression	19 (2%)
Left ventricular ejection fraction (%)	53 ± 12 (55, 5–86)
Extent of coronary artery disease	
Left main disease	386 (40%)
Single vessel	48 (5%)
Two vessel	287 (30%)
Three vessel	628 (65%)

### Operative and perfusion profile

Operative data are summarized in Table 2. A large proportion of patients were operated non-electively. The mean number of grafts reflects the fact that most patients were operated for left main or multi-vessel disease and our strong desire to achieve complete revascularization. The left internal mammary artery was used routinely to graft the left anterior descending coronary artery, regardless of age. Multiple arterial grafts were preferred in patients younger than 70 years of age. Inherent to our perfusion strategy, total heparin and protamine doses were very low compared to conventional standards, resulting in a low ACT.

**Table 2:** **Operative and perfusion data**

Variable	Percent or mean, SD (Median, range) (n = 964)
Priority of surgery	
Elective	546 (56.6%)
Urgent	348 (36.1%)
Emergent	63 (6.5%)
Emergent-salvage	7 (0.7%)
Redo surgery	14 (1.5%)
Number of vessels bypassed	2.7 ± 0.8 (3, 1–5)
Usage of arterial grafts	
Left internal mammary artery	938 (97%)
Right internal mammary artery	267 (28%)
Radial artery	125 (13%)
Cardiopulmonary bypass time (min)	95 ± 31 (93, 19–309)
Aortic cross-clamp time (min)	64 ± 21 (63, 12–169)
Lowest systemic temperature (°C)	35 ± 12 (35, 26–37)
Total heparin dose (mg/Kg)	1.9 ± 1.0 (1.7, 0.1-8.6)
Total protamine dose (mg/Kg)	1.0 ± 0.5 (0.9, 0–5.4)
Highest ACT during CPB (sec)	360 ± 326 (335, 130–999)
Lowest ACT during CPB (sec)	273 ± 42 (264, 139–524)

### Clinical outcomes

Clinical outcomes are summarized in Table 3. The observed major study outcomes were compared to the expected outcomes calculated using the STS Database risk assessment algorithms [13]. Thirty-day mortality, stroke, myocardial infarction and reoperation for bleeding were low and within the STS predicted rates. Overall the rate of postoperative renal failure was higher than expected (7.4% vs. 3.0%), although only 2.9% required dialysis. The low rates of major complications translated into short time on the respirator, and short ICU and hospital length of stay. In a subset analysis we compared the results of the two institutions. Clinical outcomes were similar between the two institutions (data not shown).

**Table 3:** **Clinical outcomes**

Outcome	Observed rate (n = 964)	STS expected rate* n = 964)
30-day mortality	14 (1.4%)	2.0%
Reoperation for bleeding	41 (4.2%)	6.0%
Postoperative stroke	9 (0.9%)	1.0%
Postoperative sternal infection	18 (1.9%)	0.5%
Postoperative renal failure	71 (7.4%)	3.0%
Postoperative MI	13 (1.3%)	
Postoperative atrial fibrillation	241 (25%)	
Time on the respirator (hours)	14 ± 29 (8, 2–432)	
ICU length of stay (hours)	74 ± 73 (48, 0–1008)	
Hospital length of stay (days)	8 ± 7 (10, 2–142)	

*Predicted outcome rate based on the American Society of Thoracic Surgeons Database risk-assessment algorithms [13].

### Bleeding and allogeneic blood transfusions

**Figure 1:**
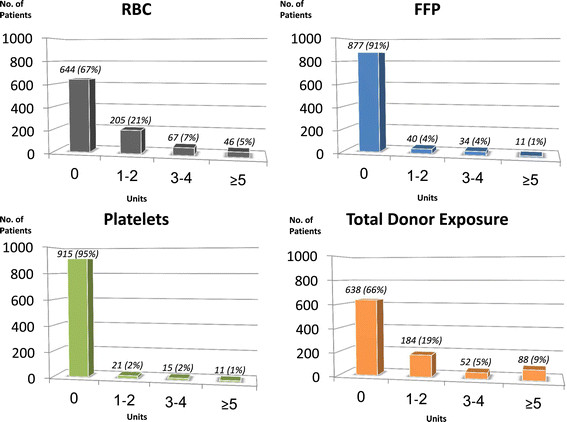
**The incidence and magnitude of allogeneic blood product transfusions.** RBC: Red blood cells; FFP: Fresh frozen plasma; PLT: Platelets.

The data on bleeding and transfusion are summarized in Table 4 and Figure 1. Median 24-hour chest tube drainage was 380 ml. Two thirds of the patients did not receive any allogeneic blood product during their admission. Those receiving blood products were exposed to a small number of donors. Discharge hematocrit was lower than the admission, but greater than 30% and without apparent adverse clinical effects.

**Table 4:** **Bleeding and transfusion data**

Variable	Percent or mean, SD (Median, range) n = 964)
24-hour chest tube drainage (ml)	439 ± 280 (380, 8–2740)
Allogeneic blood products transfusion	325 (33.7%)
Magnitude of Donor Units Transfused	
Red Blood Cells (units)	1.0 ± 1.9 (0, 0–17)
Fresh Frozen Plasma (units)	0.3 ± 1.2 (0, 0–18)
Platelets (units)*	0.3 ± 1.8 (0, 0–22)
Cryoprecipitate (units)*	0.1 ± 1.1 (0, 0–20)
Total (units)	1.7 ± 4.7 (0, 0–70)
Preoperative hematocrit (%)	40.7 ± 4.6 (41.1, 24.0-56.9)
Discharge hematocrit (%)	33.4 ± 9.3 (32.9, 22.7-28.4)

*One pack of platelets contains five units (five donors) and one pack of cryoprecipitate contains ten units (ten donors).

### Predictors of study end-points

Using multivariable logistic regression analyses we assessed the predictors of 30-day mortality and allogeneic blood transfusions. The data are summarized in Table 5. Independent predictors of mortality include left main disease and preoperative treatment with anti-arrhythmic and immunosuppressive medications. Independent predictors of allogeneic transfusions include advanced age, small body surface area and female gender. High preoperative hematocrit and LVEF were associated with reduced risk of transfusions. Advanced age and priority of surgery were not predictors of mortality. Also, the study site (Hadassah or Angers) and dual anti-platelet therapy were not predictors of mortality, or blood transfusions.

**Table 5:** **Predictors of major study endpoints in multivariable analyses**

Variable	Odd ratio	95% confidence intervals	P value
A. 30-day mortality			
Preoperative Immunosuppressive Therapy	12.98	2.38 – 70.8	0.003
Preoperative anti-arrhythmics	6.86	1.74 – 27.11	0.006
Left main disease	5.77	1.48 – 22.5	0.012
B. Allogeneic blood product transfusions			
Age	1.05	1.03 – 1.06	<0.0001
Body surface area	1.004	1.00 – 1.007	0.025
Female gender	5.07	3.26 – 7.89	<0.000
Preoperative creatinine	1.65	1.15 – 2.36	0.007
LVEF	0.97	0.95 – 0.98	<0.0001
Preoperative hematocrit	0.85	0.81 – 0.88	<0.0001

## Discussion

The mid-term results of the Syntax trial published recently re-confirmed that CABG is associated with improved survival and fewer major adverse cardiac events compared to PCI in patients with multi-vessel and complex left main coronary artery disease [1]. These advantages are particularly apparent in diabetic patients [14]. The superiority of CABG is related to a fundamental attribute of the procedure – the ability to achieve a long-lasting complete myocardial revascularization. Precise vascular anastomoses are an important determinant of short and long-term graft patency. Use of CPB provides the optimal conditions necessary to perform the anastomoses including stable hemodynamics and bloodless and motionless surgical field. Although in selected and very experienced center the goals of CABG can be achieved using the beating-heart technique, recent large scale trials studies have shown that the off-pump CABG technique is at best similar or inferior to the on-pump technique [2],[15]. However, use of CPB is associated with side effects inherent to the technology [3].

The side effects inherent to CPB originate mostly from exposure of blood to a large foreign surface, the non-physiological blood-air interphase, mandatory anti-coagulation, physical trauma to blood components, micro-embolization and hypothermia [3]. These primary processes induce activation of multiple cascades such as the intrinsic and extrinsic coagulation system, the complement and kalikrein systems as well as cellular components, leading to intense systemic inflammatory response. Altogether, these pathophysiological processes result in coagulopathy and end-organ dysfunction [3].

The comprehensive biocompatible perfusion strategy adopted by both institutions addresses many of these issues. We use membrane oxygenators and CPB circuits with modified surface coated with heparin and avoid systemic cooling, exposing the blood to a more physiologic conditions and allowing us to employ much lower level of systemic anticoagulation. Precise monitoring of anticoagulation by heparin titration curves in addition to ACT, reduces over- or under dosing of heparin and protamine. Using a closed CPB system without a cardiotomy sucker and a reservoir minimizes the deleterious effects of blood-air interphase. Reducing CPB prime decreases hemodilution. Altogether this set of modifications comprising the BPS result in a marked attenuation of the inflammatory response and coagulopathy [16]-[18], translating into improved clinical outcomes and reduced need for allogeneic blood transfusions compared to "conventional" perfusion strategy [4]-[8],[11].

The baseline profile of the current patient cohort is typical for patients referred for CABG nowadays [9]. Patients are older with multiple co-morbidities (36% were diabetics). Most patients had left main and or 3-vessel disease. Fifty eight percent had prior MI and 29% underwent at least one PCI prior to surgery. CABG was performed non-electively in 43%. Despite this high risk profile, this study demonstrates that the comprehensive BPS remains safe and effective.

The single most important outcome measure to assess quality and safety is procedural mortality. We observed a 30-day mortality of 1.4%. This is much less than the 2.0% expected by the STS Database algorithm. The STS Database risk prediction models are derived from data collected on more than two million CABG procedures. Thus, although the current study lacks a control group, the STS risk prediction models for mortality and specific complications serve as very powerful benchmarks against which our results can be compared [19].

Safety is a major concern with any technique and technology that deviates from standard practice. Despite using significantly lower level of systemic anticoagulation, we did not observe clots in the CPB circuits or experienced oxygenator malfunction. More importantly, the rates of postoperative thromboembolic complications such as CVA, MI and peripheral vascular were low and within expected, based on the STS predictive algorithms.

The BPS was not only safe, but also effective. Despite the high risk profile, clinical outcomes were very acceptable. The observed operative mortality of 1.4% was lower than the STS predicted mortality of 2.0%. Independent predictors of 30-day mortality were left main and preoperative use of immunosuppressive or anti-arrhythmic medications. Factors such as age, female gender, diabetes, renal failure, non-elective procedure and cardiopulmonary bypass time were not associated with increased risk of mortality.

The low rate of CVA (0.9%) observed in the current series despite the increased patient risk profile is encouraging. CVA has been considered as the "Achilles heel" of CABG compared with PCI even in recent studies such as Syntax [1]. We attribute the low CVA rate to several factors associated with the BPS. The elimination of the cardiotomy suction from the CPB circuit minimizes blood-air interface, substantially decreasing micro-embolization and systemic inflammatory response – well known risk factors for neurological adverse events. Using transcranial Doppler technology, we have previously shown that the number of cerebral micro-emboli was very low and associated with low rates of CVA and neurocognitive dysfunction [5]. In that same study we have also shown that the low anticoagulation protocol reduces thrombin generation during CPB, potentially reducing the risk of thromboembolic complications [5]. In fact, the incidence of other thromboembolic complications we observed such as MI (1.3%) was lower than expected by the STS prediction for our cohort. We did observe a relatively higher rate of postoperative renal failure. Although it is within the range reported recently after CABG [20], our rate is higher than the STS-predicted. We have no good explanation for this observation and plan to further investigate it.

The BPS was particularly effective in reducing bleeding and transfusion requirements. The observed rate of 4.2% for reoperation bleeding was lower than that predicted by the STS algorithm (6%) for our cohort. Median 24-hour chest tube drainage was 380 ml. Coagulopathy was very infrequently the cause for reoperation. Two thirds of patients did not receive any allogeneic blood products with an average total donor exposure of only 1.7 units. This compares favorably with reported transfusion rates of 39-70% in recent large-scale reports [21]-[24]. In contrast to these reports, advanced age, priority of surgery, preoperative dual anti-platelet therapy and CPB times were not found to be risk factors of increased transfusion. The protective effect of the BPS in patients on dual-antiplatelet therapy has been also reported by Hussaini and colleagues. [25]. It has been previously demonstrated that reoperation for bleeding, the incidence transfusion and the magnitude allogeneic blood transfusion are all independent risk factors of adverse outcomes after cardiac surgery [21]-[24]. We believe that the favorable clinical outcomes observed in the current study are strongly related to reduced bleeding and very low rate of allogeneic blood product utilization. Finally, reduced rates of major complications, bleeding and transfusion requirements translated into short respiratory support times, short ICU and total hospital lengths of stay.

There are prior studies that found limited value and small clinical benefit to biocompatible CPB circuits [26]. A careful look at these studies reveals that the perfusion technique practiced by different authors was highly variable, missing many essential components of our comprehensive BPS. Examples include using open (versus closed) systems, using CPB circuits that are not tip-to-tip coated, inclusion of cardiotomy suction and conventional levels of systemic anticoagulation. We strongly believe that in order to be truly biocompatible and clinically effective, our BPS should be implemented as a whole. Eliminating one or more components might have a major adverse impact on its biocompatibility.

This study was not designed to evaluate the cost-effectiveness of the BPS. However, it seems that the apparent clinical benefits may very well counter-balance the incremental cost of the technology. LaPar and colleagues recently demonstrated the substantial cost saving achieved by reducing blood transfusions after isolate CABG [24]. This aspect mandates further investigation in our institutions.

Study limitations. Although the data were entered prospectively, this is a retrospective study with the inherent limitation of a selection bias. However, the study cohort is large, consecutive and typical of contemporary practice, allowing meaningful multivariable analyses of outcomes. Although both centers employed a similar perfusion strategy, non-measured differences in practice between institutions might have affected outcomes. Finally, there is no control group. However, the use of the STS risk prediction models provides benchmarks against which the outcomes of this series can be compared. In fact, given our previous prospective randomized trials and the results of the current study, it would be difficult to ethically justify such a trial.

## Conclusions

Despite the study limitations, we conclude that the our data suggest that in a contemporary cohort of patients undergoing CABG, the use of BPS is safe and effective. It is associated with excellent clinical outcomes and reduced allogeneic blood transfusions.

## Authors’ original submitted files for images

Below are the links to the authors’ original submitted files for images. Authors’ original file for figure 1

## Abbreviations

ACT: Activated clotting time
BPS: Biocompatible perfusion strategy
CABG: Coronary artery bypass grafting
CPB: Cardiopulmonary bypass
CVA: Cerebrovascular accident
FFP: Fresh frozen plasma
ICU: Intensive care unit
LVEF: Left ventricular ejection fraction
MI: Myocardial infarction
PCI: Percutaneous coronary intervention
PLT: Platelets
RBC: Red blood cells
STS: Society of Thoracic Surgeons
