# Supplementary figures and images for: Safety and efficacy of biocompatible perfusion strategy in a contemporary series of patients undergoing coronary artery bypass grafting – a two-center study
Source: J Cardiothorac Surg. 2014 Dec 18;9:196. doi: 10.1186/s13019-014-0196-3 (PMC4274677; doi:10.1186/s13019-014-0196-3)

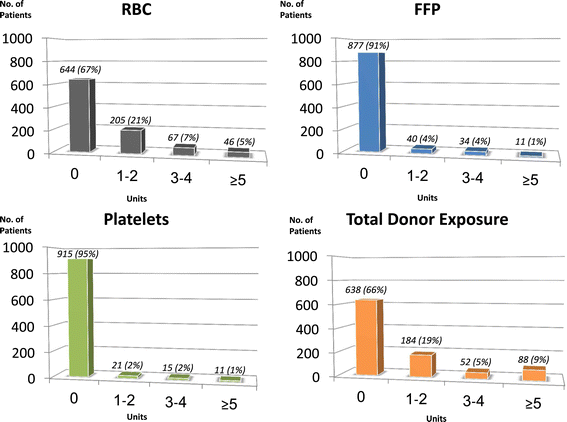

Supplement: Supplementary file 1 — Authors’ original file for figure 1 [file 13019_2014_196_MOESM1_ESM.gif]
